# Phase I study of TP300 in patients with advanced solid tumors with pharmacokinetic, pharmacogenetic and pharmacodynamic analyses

**DOI:** 10.1186/1471-2407-12-536

**Published:** 2012-11-21

**Authors:** D Alan Anthoney, Jay Naik, Iain RJ MacPherson, Donna Crawford, John M Hartley, Janet A Hartley, Tomohisa Saito, Masaichi Abe, Keith Jones, Masanori Miwa, Christopher Twelves, TRJ Evans

**Affiliations:** 1 St James Institute of Oncology, University of Leeds & Leeds Teaching Hospitals Trust, Leeds LS9 7TF, United Kingdom; 2 University of Glasgow, Beatson West of Scotland Cancer Centre, Glasgow, G12 OYN, United Kingdom; 3 UCL Cancer Institute, Paul O’Gorman Building, University College London 72 Huntley Street, London, WC1E 6BT, United Kingdom; 4 Chugai Pharmaceutical Co., Ltd, Nihonbashi Muromachi 2-1-1, Chuo-ku, Tokyo, 103-8324, Japan; 5 Chugai Pharmaceuticals Europe Ltd. Turnham Green, London, W4 1NN, United Kingdom

**Keywords:** Pharmacodynamics, Pharmacogenomics, Pharmacokinetics, Phase I study, Safety profile, Topoisomerase-I inhibitor

## Abstract

**Background:**

A Phase I dose escalation first in man study assessed maximum tolerated dose (MTD), dose-limiting toxicity (DLT) and recommended Phase II dose of TP300, a water soluble prodrug of the Topo-1 inhibitor TP3076, and active metabolite, TP3011.

**Methods:**

Eligible patients with refractory advanced solid tumors, adequate performance status, haematologic, renal, and hepatic function. TP300 was given as a 1-hour i.v. infusion 3-weekly and pharmacokinetic (PK) profiles of TP300, TP3076 and TP3011 were analysed. Polymorphisms in CYP2D6, AOX1 and UGT1A1 were studied and DNA strand-breaks measured in peripheral blood mononuclear cells (PBMCs).

**Results:**

32 patients received TP300 at 1, 2, 4, 6, 8, 10, 12 mg/m^2^. MTD was 10 mg/m^2^; DLTs at 12 (2/4 patients) and 10 mg/m^2^ (3/12) included thrombocytopenia and febrile neutropenia; diarrhoea was uncommon. Six patients (five had received irinotecan), had stable disease for 1.5-5 months. TP3076 showed dose proportionality in AUC and C_max_ from 1–10 mg/m^2^. Genetic polymorphisms had no apparent influence on exposure. DNA strand-breaks were detected after TP300 infusion.

**Conclusions:**

TP300 had predictable hematologic toxicity, and diarrhoea was uncommon. AUC at MTD is substantially greater than for SN38. TP3076 and TP3011 are equi-potent with SN38, suggesting a PK advantage.

**Trial registration:**

EU-CTR2006-001345-33

## Background

Inhibition of topoisomerase-I (Topo-1) is a clinically proven treatment strategy for many cancers [1]. Irinotecan hydrochloride is the most widely used topoisomerase-I inhibitor, approved for the treatment of patients with colorectal cancer previously treated with 5-fluorouracil [2]. It also has activity against a wide range of other cancers (eg. glioma, gastric, non small cell lung and pancreatic cancers), either as a single agent or in combination [3-6]. Irinotecan has, however, a number of properties that limit its usefulness. It is metabolized enzymatically by carboxylesterase 2 (CES2), predominantly within the liver, to SN-38 (a significantly more potent Topo-1 inhibitor). This conversion shows considerable inter-individual variability, resulting in a wide range of systemic SN-38 exposure for a given dose that may influence the efficacy and toxicity of irinotecan. Clinically, the use of irinotecan is limited by diarrhoea and neutropenia with potential impact on dose intensity as well as patient acceptability; low activity of the SN-38 metabolising enzyme UGT1A1 is associated with a greater risk of diarrhoea and myelosuppression [6], and in 2005 the US FDA recommended irinotecan dosing be modified in patients carrying the UGT1A1*28 polymorphism [2,7]. The development of Topo-1 inhibitors not subject to such pharmacogenomic variability might, therefore, enhance the clinical efficacy of this class of agents.

**Figure 1:**
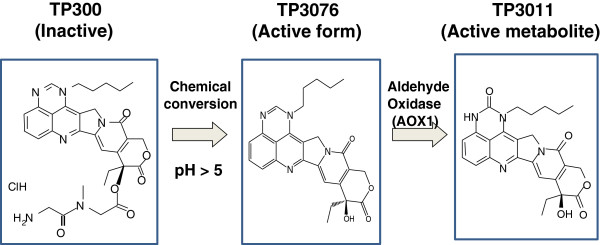
The fate of TP300, active form (TP3076) and its metabolite (TP3011).

TP300 (glycine,glycyl-N-methyl-,(9S)-9-ethyl-9,10,13,15-tetrahydro-10,13-dioxo-1-pentyl-1H,12H-pyrano[3'',4'':6′,7′]indolizino[2′,1′:5,6]pyrido[4,3,2-de]quinazolin-9-yl ester, hydro-chloride) has been developed as a water soluble prodrug of the Topo-1 inhibitor TP3076, and its active metabolite, TP3011, both of which are equipotent to SN38 in terms of Topo-1 inhibition (Figure 1) [8]. TP300 has activity at nanomolar concentrations across a range of tumour types *in vitro* and, unlike SN38, appears active in tumours over-expressing the breast cancer resistance protein [BCRP] [8]. In man, TP300 is converted non-enzymatically to TP3076, then metabolized to TP3011 by aldehyde oxidase 1 (AOX1; Figure 1) [9]. TP3011 and TP3076 are equipotent as Topo-1 inhibitors, with IC_50_ in the sub-nanomolar range HCT-116 colorectal cancer cells *in vitro*[10]. Importantly, TP3076 lacks a phenolic-OH group in its structure such that it cannot be glucuronidated in the same way as irinotecan and also should not be influenced to any great extent by polymorphisms in the UGT1A1 gene. There should, therefore, be less inter-individual variation in activation and toxicity with TP300 than with irinotecan; specifically, it would be expected that severe diarrhoea should not be an issue.

The primary objectives of this Phase I first in man study of TP300 in patients with advanced solid tumours, were to establish the maximum tolerated dose (MTD), dose-limiting toxicity (DLT), and recommended Phase II dose of TP300 but also incorporated pharmacokinetic, pharmacogenomic and pharmacodynamic evaluation.

## Methods

### Patients and eligibility criteria

This was a Phase I, open-label, non-randomized, two center dose-escalation study, conducted in accordance with the ICH GCP and approved by each participating institution’s Research Ethics Committee. All patients provided written informed consent.

Eligible patients were ≥ 18 years old, with histologically or cytological confirmed advanced solid malignancies who were refractory to standard therapies or for whom there was no effective standard therapy, and with Eastern Cooperative Oncology Group (ECOG) performance status ≤ 2. Further criteria for inclusion included adequate bone marrow function (neutrophil count ≥ 1.5 x10^9^, platelet count ≥ 1 x10^9^, and haemoglobin≥10.0 g/dL) and adequate hepatic (serum bilirubin ≤ 1.5 x upper limit of normal [ULN], alanine amino transferase (ALT) and aspartate amino transferase (AST) ≤ 2.0 x ULN) and renal (serum creatinine ≤ 1.5 x ULN) function.

Standard Phase I trial exclusion criteria included exposure to prior cytotoxic chemotherapy, extended field radiotherapy or surgery within 4–6 weeks before the start of the study; presence of severe concomitant medical illness; and the presence of symptomatic brain metastases. A history of severe or life-threatening drug allergy or hypersensitivity to camptothecin derivatives and diarrhoea (excess of 2–3 stools/day above normal frequency within 2 weeks prior to the start of the study) were additional exclusion criteria.

### Treatment and dose escalation

TP300 was given as a 1-hour intravenous (i.v.) infusion, by peripheral venous catheter, every 3 weeks.

TP300 sterile concentrate solution for intravenous infusion was supplied in vials containing 5 mL solution at a concentration of 4 mg/mL of the free base active ingredient. Other ingredients were glycine, sodium chloride, hydrochloric acid and water for injection. Before infusion, each vial was diluted in 0.9% sodium chloride for infusion. Infusion bags used for the different dosages were < 2.0 mg/m^2^ TP300 50 mL and at other dosages 100 mL. pH was less than 2, so care was taken with the site of administration, looking for any evidence of local irritation.

The starting dose of 1 mg/m^2^ was derived from single and repeat dose toxicity studies and represented one sixth of 0.3 mg/kg (6 mg/m^2^), a dose tolerated without evidence of serious, irreversible or life-threatening toxicity in the most sensitive of the two species tested. Doses were doubled in subsequent cohorts until grade ≥2 toxicity was observed, whereupon a modified Fibonacci dose escalation scheme was used. In each cohort the first patient was required to complete 1 cycle before subsequent patients were entered. Intra-patient dose-escalation was not allowed.

Three patients were planned per cohort, with up to 3 added if dose limiting toxicity (DLT) was observed in the initial group, and an expanded cohort at the maximum tolerated dose (MTD). The MTD was the dose level below that at which ≥ 2 out of 3–6 patients experienced a DLT. Radiologic assessment of disease was performed every 2 cycles. Patients could remain on treatment if there was evidence of clinical benefit but were withdrawn from the study upon clinical or radiologic progression, unacceptable toxicity or withdrawal of consent.

### Evaluation of toxicity

Toxicity was assessed weekly and graded using the National Cancer Institute Common Toxicity Criteria (CTCAE) version 3.0. DLT was defined as the occurrence of any of the following adverse events: grade 4 thrombocytopenia; febrile neutropenia or grade 4 neutropenia > 5 days duration; grade 4 diarrhoea not reduced to grade 1 within 2 days of appropriate therapy; other gastro-intestinal toxicities (e.g. vomiting, nausea, stomatitis) ≥ grade 3 and not reduced to grade 1 within 2 days of appropriate therapy; any other non-haematologic toxicities ≥ grade 3 (excluding alopecia).

### Pharmacokinetic assessments

Venous blood (3 mL) was taken into sodium heparin tubes containing 30 μL of 46% citric acid, centrifuged 1500*g* at 4°C for 10 min and 1 mol/L hydrochloric acid was added (1:10) to plasma from each subject at 10 time points during cycle 1 (pre dose, then 1, 1.25, 1.5, 2, 3, 5, 8, 24, 48 hours after the start of administration) and stored at −70°C. Samples were processed by extraction of protein using addition of organic solvent containing internal standards and assayed by LC-MS/MS with a robust, sensitive and validated method for the simultaneous determination of a novel topoisomerase 1 inhibitor CH0793076 (TP3076), the prodrug CH4556300 (TP300), and the active metabolite CH0793011 (TP3011) [9]. All plasma had been acidified during collection to avoid the pH-based degradation of TP300 and to shift the equilibria of TP3076 and TP3011 between the lactone and carboxylate forms towards the lactone forms. After the plasma proteins were precipitated with methanol:acetonitrile:HCl 1M (50:50:1, v:v:v) containing stable isotopic internal standards, the analytes were trapped on an Xterra MS C18 column (10×2.1 mm i.d., 5 μm) and separated on a Gemini C18 column (50×2.0 mm i.d., 5 μm) using column-switching liquid chromatography. Electrospray ionization in the positive-ion mode and multiple reaction monitoring were used to quantify the analytes with transitions m/z 587.2>441.2 for TP300, 459.1>415.2 for 3076, and 475.1>361.1 for 3011. The inter- and intra-day precisions were below 12%, and the accuracy was between −16% and 16% at the lower limit of quantitation (LLOQ) and between −11% and 14% at the other quality controls. The LLOQs of TP300, TP3076, and TP3011 were 0.8, 0.04, and 0.04 ng/mL, respectively.

Pharmacokinetic parameters derived from plasma concentration-time data of TP3076 or TP3011 by non-compartmental methods using WinNonlin (version 5.1; Pharsight Corp), included maximal plasma concentration (C_max_) and time of C_max_ (T_max_), apparent plasma elimination half-life (t_1/2_), and the area under the curve (AUC). The urinary excretion ratio (f_e_) of TP3076 and TP3011 was calculated from the urinary concentration and volume up to 48 hours after administration, and the administration dose. The linearity of C_max_ and AUC was determined with linear regression, analysis of variance and power model analysis. The sum of the AUCs of TP3076 and TP3011 was plotted against percentage (%) fall in nadir neutrophil count to explore the relationship between exposure and myelosuppression as a measure of anti-proliferative effect and a sigmoid E_max_ model fitted to the data:

(1) $$\text{Effect}=\frac{{\left(AUC_{TP3076}+AUC_{TP3011}\right)}^{\gamma }}{{\left(AUC_{TP3076}+AUC_{TP3011}\right)}^{\gamma }+EAU{C_{50}}^{\gamma }}$$

where γ is the Hill equation constant and EAUC_50_ is the AUC associated with 50% of the maximal effect.

### Pharmacogenomic analysis

Blood samples for pharmacogenomic analysis were collected and all samples anonymised for subsequent storage, assay and analysis. The impact on C_max_ and AUC of genetic polymorphisms of CYP2D6, which is assumed to make some contribution to the hydroxylation of TP3076, but not TP3011, AOX1, which metabolises TP3076 to TP3011, and UGT1A1, which metabolises SN-38 to its glucuronide, but is not believed to influence TP300 metabolism were explored. The analysis was performed with the invader method or polymerase chain reaction (PCR)-invader method for single nucleotide polymorphisms (SNPs) of CYP2D6 and UGT1A1 *28 and with the long-PCR method for deletion of CYP2D6 and UGT1A1 gene. AOX1 SNPs were analysed by a direct sequencing method. Genomic DNAs were extracted from frozen peripheral blood of 31 subjects using an automated DNA extractor BioRobotMDx and commercial DNA purification kits (Qiagen). Quality and quantity of the DNAs were checked by the measurement of absorbencies at 260 nm and 280 nm. Primers were designed to amplify all the 35 exons of *Aldehyde oxidese* 1 gene (NCBI accession No. NM_001159) including some intronic flanking regions. The specificity of PCR conditions was confirmed by the agarose gel electrophoresis. Amplicons were prepared twice for every exons. The amplicons were subsequently treated with ExoSAP-IT (GE Healthcare) followed by the reactions with a cycle sequencing kit (BigDye Terminator v3.1, Applied Biosystems). The fragments obtained were purified using X-Terninator purification kit (Applied Biosystems) and analysed on an automated DNA sequencer (3730xl DNA Analyzer, Applied Biosystems). The resulted sequences were compared against the reference sequence using the variant reporter software (Applied Biosystems).

### Pharmacodynamic analysis

Analysis of the ability of TP300 to induce DNA strand breaks was performed on peripheral blood mononuclear cells (PBMCs), pre-dose, 1, 3 and 24 hours post cycle 1. A validated single cell gel electrophoresis (comet) assay was used to assess DNA single-strand breaks [11]. An average of 50 PBMC cells/time-point were analysed and the tail moment (TM) calculated, as the product of the percentage of DNA in the comet tail and the distance between head and tail distributions; higher TM values reflect greater DNA strand breakage. Statistical analyses were not performed due to the limited number of subjects/samples per cohort.

## Results

### Patient characteristics

Thirty two patients were recruited between September 2006 and October 2008. TP300 doses were 1, 2, 4, 6, 8, 10, 12 mg/m^2^. Of the 32 patients, 21 received two cycles of TP300, five received three to seven cycles. Patient characteristics are shown in Table 1.

**Table 1:** Demographic and baseline characteristics

**Demographic data**		
Variable		Total
		N=32
Age (years)		31-72 (58.0)
Number of regimens of previous chemotherapy		1-4 (2)
Sex	Male	20 (62%)
	Female	12 (38%)
ECOG performance status	0	8 (25%)
	1	20 (63%)
	2	4 (13%)
Site of primary cancer	Breast	1 (3%)
	Colon	6 (19%)
	Lung	1 (3%)
	Pancreas	2 (6%)
	Pleura	1 (3%)
	Rectum	7 (22%)
	Soft tissue	2 (6%)
	Stomach	4 (13%)
	Other	8 (25%)

Continuous data: Minimum-Maximum (Median).

### Toxicity

Toxicity from TP300 was predominantly haematologic with neutropenia the DLT. Minimal toxicity was observed at doses up to and including 8 mg/m^2^_._ Two/four patients dosed at 12 mg/m^2^ experienced DLT: grade 4 febrile neutropenia (5 days duration) and grade 4 neutropenia (15 days). As 8 mg/m^2^ had been well tolerated, the intermediate dose of 10 mg/m^2^ was explored in 12 patients, and 3 experienced a DLT: grade 3 febrile neutropenia (9 days); grade 4 neutropenic sepsis (8 days) with concomitant grade 4 thrombocytopenia (7 days); and grade 4 uncomplicated neutropenia (7 days). No patient received growth factor support during neutropenic episodes. Six patients, who had experienced DLT, on subsequent recovery, continued TP300 dosed at the previous dose.

Non-haematologic toxicity was generally mild and self-limiting and no patient experienced cholinergic effects. A summary of grade ≥ 3 toxicities is shown in Table 2. Specifically, only 8 patients developed diarrhoea, grade 2 at worst and arising on average 9.5 days (range 1 to 27 days) after TP300 treatment; only one patient had concomitant dose-limiting myelosupression. There were no other ≥ grade 3 gastrointestinal toxicities.

**Table 2:** Summary of suspected treatment related adverse events

	**Summary of adverse events occurred within cycle 1**	
	All grade N = 32 No. (%)	Grade 3 N = 32 No. (%)
Diarrhea	8 ( 25)	0 (0)
Nausea	13 ( 41)	0 (0)
Vomitting	9 ( 28)	0 (0)
Anaemia	6 ( 19)	2 ( 6)
Neutropenia	6 ( 19)	6 ( 19)
Leukopenia	3 ( 9)	3 ( 9)
Thrombocytopenia	3 ( 9)	3 ( 9)
Febrile neutropenia	2 ( 6)	2 ( 6)
Lethargy	12 ( 38)	4 ( 13)
Syncope	1 ( 3)	1 ( 3)
Chills	2 ( 6)	1 ( 3)
Hepatorenal failure	1 ( 3)	1 ( 3)
Neutropenic sepsis	1 ( 3)	1 ( 3)
Back pain	1 ( 3)	1 ( 3)
Dyspnoea	1 ( 3)	1 ( 3)

TP300 was discontinued due to toxicity in 4 patients, but there were no treatment-related deaths. TP300 dose was modified at cycle 2 in 4 patients; from 12 to 8 mg/m^2^ 1 patient, from 10 to 8 mg/m^2^ 2 patients, and from 6 to 4 mg/m^2^ 1 patient. Five cycles were delayed in 3 patients due to disease-related morbidity (3), gastroenteritis (1), and anaemia (1).

### Antitumour activity

There were no complete or partial responses as determined by RECIST. However, six patients had stable disease as their best response (1 each at 4, 8 and 12 mg/m^2^ and 3 at 10 mg/m^2^) lasting between 1.5-5 months of whom five had previously been treated with irinotecan. One patient with metastatic gastric adenocarcinoma (4 mg/m^2^) had a clinically significant improvement of malignant ascites and stabilization of established peritoneal metastases over 7 cycles.

### Pharmacokinetic analyses

**Figure 2:**
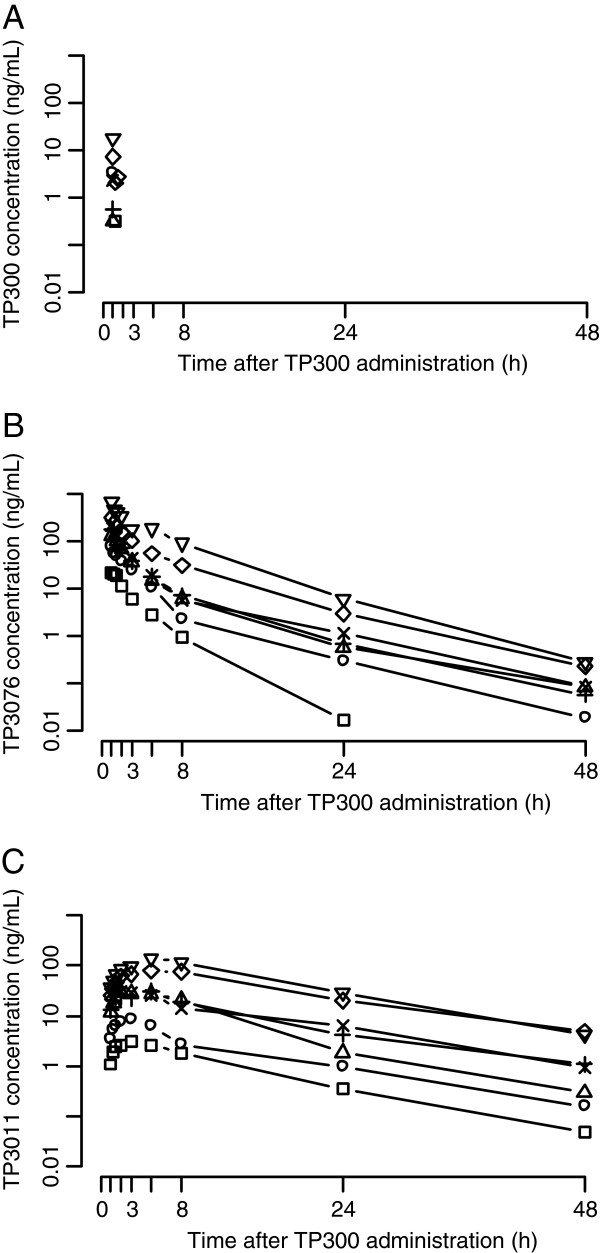
**Time-plasma concentration profile of TP300, TP3076 and TP3011. A**: The plasma concentration profile of TP300. **B**: The plasma concentration profile of TP3076. **C**:The plasma concentration profile of TP3076. Square:1 mg/m^2^, Circle:2 mg/m^2^, Triangle (point up):4 mg/m^2^, Cross:6 mg/m^2^, X:8 mg/m^2^, Diamond:10 mg/m^2^, Triangle (point down):12 mg/m^2^.

**Figure 3:**
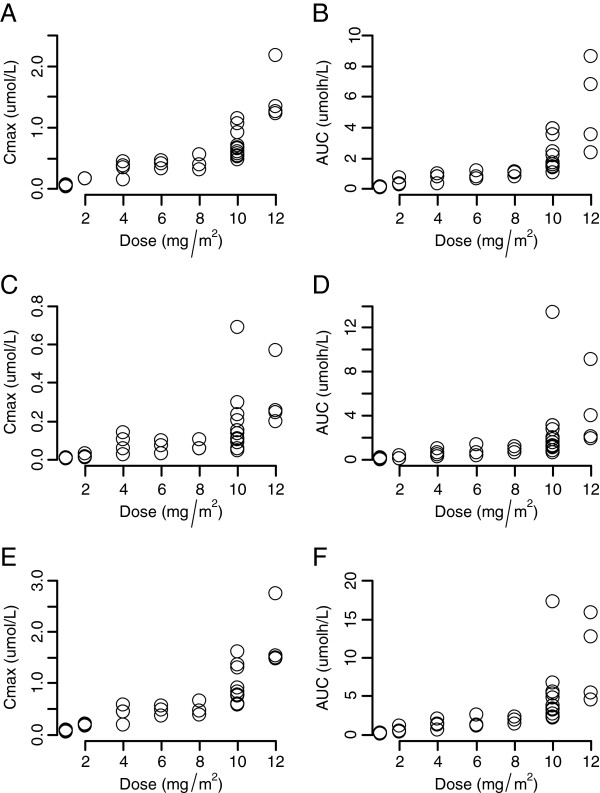
**Scatterplot of exposure against dose. A**: The scatterplot of TP3076 Cmax. **B**: The scatterplot of TP3076 AUC. **C**: The scatterplot of TP3011 Cmax. **D**: The scatterplot of TP3011 AUC. **E**: The scatterplot of the sum of TP3076 and TP3011 Cmax. **F**: The scatterplot of the sum of TP3076 and TP3011 AUC.

The plasma concentration-time profiles of TP3076 and TP3011 are shown in Figure 2 and a summary of pharmacokinetic parameters in Table 3. TP300 rapidly disappeared and all measured concentrations 1.5 h after dose were below the limit of the quantification. Plasma TP300 concentration 1 h after administration was obtained only at the doses of 2, 8, 10 and 12 mg/m^2^ and the mean concentration was 3.48, 2.34, 7.37 and 18.4 ng/mL, respectively. Meanwhile, T_max_ for TP3076 was at the end of infusion (1 hour) and that of the metabolite TP3011 was at 3–5 hours. Urinary excretion ratios of TP3076 and TP3011 were low and represented at most 6% at the highest dose. The C_max_ and AUC of TP3076 and TP3011 increased proportionately up to 10 mg/m^2^ TP300 (Figure 3) and inter-patient variability was small.

**Table 3:** Summary of pharmacokinetic parameters of TP3076 and TP3011

**TP3076**	**Dose (mg/m**^**2**^**)**	**1**	**2**	**4**	**6**	**8**	**10**	**12**
**Parameter**	**N**	**3**	**3**	**3**	**3**	**3**	**12**	**4**
C_max_ (ng/mL)	Mean	26.1	76.9	152	185	196	326	688
	S.D.	7.46	3.65	56.4	29.8	56.1	99.4	209
AUC (ng*h/mL)	Mean	52.9	209	335	405	451	1000	2450
	S.D.	8.26	103	130	120	87.8	476	1330
t_max_ (h)	Mean	1.26	0.966	1.02	1.01	1.10	1.07	0.983
	S.D.	0.242	0.0289	0.168	0.0587	0.137	0.111	0.0236
t_1/2_ (h)	Mean	2.35	3.73	7.04	5.97	6.22	5.14	4.93
	S.D.	0.990	1.33	3.30	0.951	0.979	1.08	0.535
f_e_ (%)	Mean	2.45	2.87	1.31	1.78	1.71	3.09	6.33
	S.D.	1.14	0.769	0.830	1.46	1.02	2.52	1.20
TP3011	Dose (mg/m^2^)	1	2	4	6	8	10	12
Parameter	N	3	3	3	3	3	12	4
C_max_ (ng/mL)	Mean	3.13	9.12	38.3	32.5	34.2	89.5	150
	S.D.	1.97	4.93	23.5	16.4	12.0	82.3	80.5
AUC (ng*h/mL)	Mean	35.1	94.0	284	379	427	1300	2030
	S.D.	24.8	77.2	140	259	114	1640	1590
t_max_ (h)	Mean	3.21	2.66	2.58	3.84	3.11	4.59	5.27
	S.D.	1.74	0.601	0.811	1.98	1.83	1.62	2.06
t_1/2_ (h)	Mean	6.92	6.56	7.18	10.0	9.52	8.60	7.41
	S.D.	3.15	2.66	2.87	2.87	1.72	1.95	1.13
f_e_ (%)	Mean	0.792	0.739	0.796	0.593	0.594	1.10	1.99
	S.D.	0.676	0.279	0.633	0.492	0.385	0.987	1.59

**Figure 4:**
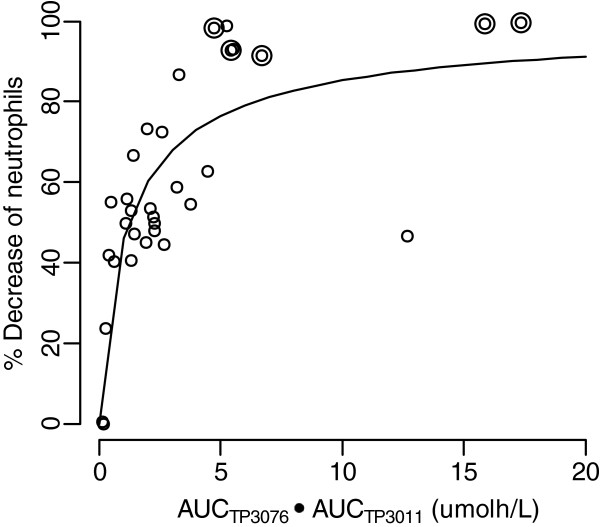
**Scatter plot of 1-Nadir/Pre-observation against AUC of TP3076+TP3011. **Circle: Subject without DLT Double circle: Subject with DLT Solid line: Curve with sigmoid E_max _model.

Pharmacokinetic analyses revealed a strong relationship between exposure to the metabolites of TP300 and falls in the neutrophil count. Figure 4 shows a scatter plot of 1-nadir/pre-observation neutrophils in cycle 1 against the total AUC of TP3076 plus TP3011 with a sigmoid E_max_ curve fitted. EAUC_50_ was 1.31 and γ was 0.862. The fall in neutrophil count was related to total AUC of TP3076 plus TP3011. All 5 patients with haematologic DLT were amongst the 9 who had a total TP3076 and TP3011 AUC of approximately 4.5 μmol*h/L or more.

### Pharmacogenetic analyses

**Figure 5:**
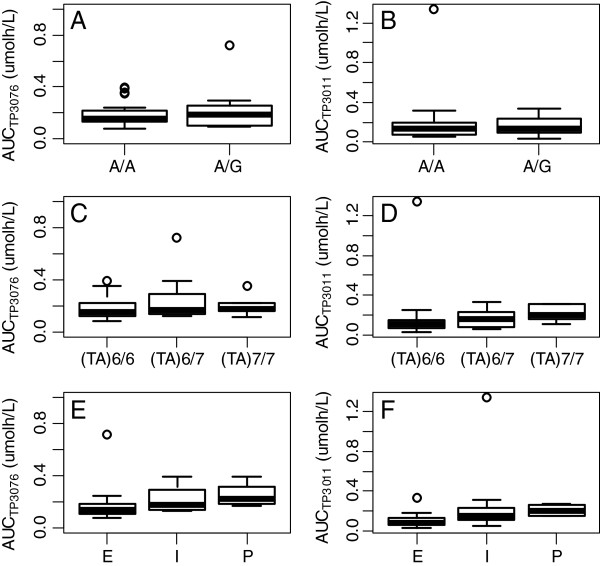
The boxplot of AUC by genotype; AOX1(c3404 A>G), UGT1A1*28 or CYP2D6. A:The boxplot of TP3076 AUC by AOX1(c3404 A>G). **B**:The boxplot of TP3011 AUC by AOX1(c3404 A>G). **C**:The boxplot of TP3076 AUC by UGT1A1 *28. **D**:The boxplot of TP3011 AUC by UGT1A1 *28. **E**:The boxplot of TP3076 AUC by CYP genotype. **F**:The boxplot of TP3011 AUC by CYP genotype. **E**: Extensive metabolizer. I:Intermediate metabolizer. P:Poor metabolizer.

The C_max_ and AUC of TP3076 or TP3011 were categorized with respect to CYP2D6, AOX1 and UGT1A1 genotypes and box plots were prepared (Figure 5). The (TA)6/6 (n=16), (TA)6/7 (n=10) and (TA)7/7 (n=5) genotypes of UGT1A1*28 were identified, but there was no apparent significant difference in exposure among these genotypes. A/A (n=23) and A/G (n=8) genotypes of AOX1 (c3404A > G) were observed; again, there was no apparent significant difference of exposure among these genotypes. The CYP2D6 phenotype was divided into extensive (neither CYP2D6 *3 or *4 mutation; n=13), intermediate (heterozygous *3 or *4 mutation; n=14) and poor (homozygous *3 or *4 mutation; n=4) metabolizers [12,13]. There appeared to be a slight reduction in exposure to TP3076 in the extensive metabolisers, with a corresponding slight decrease in TP3011 exposure.

### Pharmacodynamic analyses

**Figure 6:**
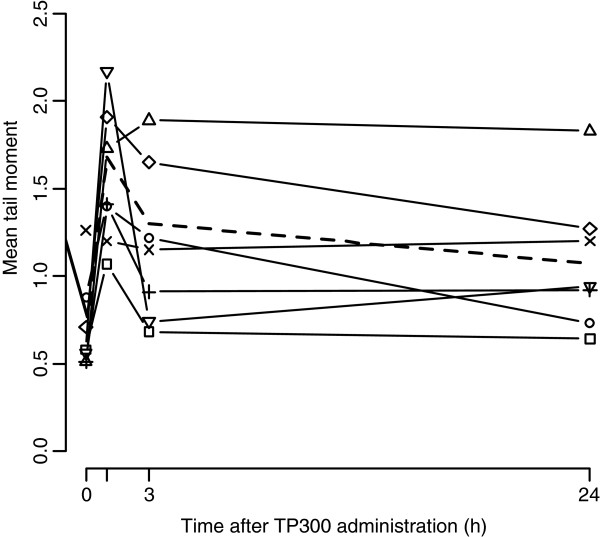
**Mean tail moment of COMET assay result over time by cohort. **Square:1 mg/m^2^, Circle:2 mg/m^2^, Triangle (point up):4 mg/m^2^, Cross:6 mg/m^2^, X:8 mg/m^2^, Diamond:10 mg/m^2^, Triangle (point down):12 mg/m^2^ Dashed line: Overall mean.

Full comet profiles (pre-dose, 1, 3, 24 hours post first dose) were available in 29 patients. The overall pre-treatment study mean tail moment (TM) across all doses was 0.69; compared to 1.65 at 1 hour, indicating approximately 2-fold increase of DNA strand breaks. Although there was no clear relationship between TP300 dose and the extent of strand breaks, the highest two doses (10 and 12 mg/m^2^) were associated with greater DNA damage (Figure 6). The mean TM was generally lower at 3 hours compared with 1 hour, with little further change at 24 hours.

## Discussion

This Phase I study demonstrates that the novel topoisomerase-I inhibitor TP300 has a good tolerability profile, and achieved several key aims that were central to its design. More specifically, as an inactive pro-drug it is rapidly converted to the active form TP3076, then metabolized to TP3011 in a consistent manner, not influenced by genetic polymorphisms. The likelihood of unpredictable, severe diarrhoea is diminished by the absence of the variable glucuronidation associated with SN-38. As predicted, TP300 does not cause acute diarrhea, which results from acetylcholine esterase inhibition [8]. Target interaction with the induction of DNA strand breaks was shown.

The main toxicity of TP300 was haematologic with neutropenia and, to a lesser extent thrombocytopenia, being dose limiting. In general, neutropenia was short lived; no patient received G-CSF support (acutely/prophylactically). At the maximum achievable dose, 12 mg/m^2^, grade 4 haematologic toxicity was observed (2/4 patients). As there had been no grade 3/4 haematologic toxicity at 8 mg/m^2^, 10 mg/m^2^ was explored in 12 patients. At 10 mg/m^2^ 3 patients experienced grade 4 haematologic toxicity and although generally well tolerated, there was a risk of short lived but significant neutropenia and thrombocytopenia. The recommended Phase II starting dose is 8 mg/m^2^, escalating to 10 mg/m^2^ on subsequent cycles, if initial treatment is well tolerated.

In marked contrast to irinotecan, gastrointestinal toxicity was in general mild, with no diarrhoea greater than grade 2. Likewise, there were no acute cholinergic reactions with its associated early diarrhoea [8,14,15]. This validates the design of TP300 as acute cholinergic reactions are associated with the 4-piperidinopiperidine moiety at the 10-position of irinotecan [16], not found in TP300.

Pharmacokinetic data confirm that TP300 is rapidly converted in plasma to the active metabolite TP3076, supporting a pH dependent chemical change occurring at physiological conditions. Hepatic aldehyde oxidase converts TP3076 to a further metabolite TP3011, which reaches maximum concentrations 3–5 hours after the end of infusion, and also has potent topoisomerase-I inhibitory activity. Pharmacogenetic analysis of aldehyde oxidase genotype, which was reported to affect the azathioprine-treated outcome [17], did not show any effect on exposure to either TP3076 or TP3011. Glucuronidated TP3076 was not detected, reflecting UGT1A1 variant status had no influence on exposure to either TP3076 or TP3011. These pharmacokinetic data reflect the design strategy. There may be a small effect of CYP2D6 metaboliser genotype on exposure to TP3076, and consequently TP3011. The AUC of TP3076 and TP3011 were linearly proportional up to 10 mg/m^2^, but at 12 mg/m^2^ there was greater inter-patient variability.

There was a strong relationship between the combined total AUC of TP3076 and TP3011 and the nadir neutrophil count, with an AUC of ≥ 4.5 hr.μmol/L generally correlating with a more significant neutrophil fall, specifically 5 of 8 patients (62.5%) with an AUC above this value experienced dose limiting neutropenia. With a 3-weekly dosing regimen of irinotecan, at 350 mg/m^2^, DLTs occur at AUCs of the active component, SN-38, of 1.5 μmol.h/L [18], SN-38 AUC is variable, influenced by UGT polymorphism. The active components of TP300 (TP3076 and TP3011) are equipotent to SN38 as Topo-1 inhibitors [8] and are not influenced by UGT polymorphisms. This means, therefore, that the combined AUC of the active components of TP300 is approximately 3-fold greater than that of SN-38, with reduced inter-individual variability indicating greater predictability of toxicity.

The comet assay demonstrated a consistent pattern with increased PBMC DNA strand breaks 1 hour after the end of infusion, generally falling by 3 hours. A similar pattern with modest and transient appearance of strand breaks was seen with temozolomide [19]. Although there were more strand breaks at higher TP300 doses, this was less clear than the relationship between pharmacokinetic exposure and neutrophil fall. However the comet data give valuable proof-of-principle that TP300 is damaging DNA, but the semi-quantitative nature does not allow a biologically optimal dose of TP300 to be identified. Without published data on DNA strand breaks in patients treated with irinotecan, a direct comparison with TP300 cannot be made. A more relevant pharmacodynamic endpoint in future may be to measure DNA strand breaks in tumour cells.

There were no objective tumour responses. However, one patient with metastatic gastric adenocarcinoma, bilateral ovarian metastases and malignant ascites requiring paracentesis prior to treatment had complete resolution of her ascites although there was no radiological change in the size of the metastatic deposits whilst receiving 7 cycles of TP300. A further 5 patients with metastatic adenocarcinoma of the colon or rectum had stable disease as their best response, with 2 having disease control for at least 4 cycles. All of these patients had received irinotecan as part of their previous chemotherapy with the patients having the most durable disease control on TP300 having had a prior response to irinotecan chemotherapy.

Topo-1 inhibitors remain clinically important in the treatment of patients with cancer. TP300 has advantages over other agents in this class in terms of tolerability and the predictability of its principle toxicity, myelosuppression. Along with the apparent PK advantage of TP300 over irinotecan, biological activity evidenced by DNA strand breaks, and preliminary evidence of clinical activity, these data warrant further evaluation of TP300.

## Conclusions

TP300 has biological activity as evidenced by DNA strand break, with a clear relationship between exposure and neutropenia, a toxicity profile superior to that of irinotecan, and preliminary evidence of clinical activity. The 10 mg/m^2^ TP300 dose level was tolerable, but the frequency of grade 4 neutropenia during cycle 1 led to the recommended Phase II dose being 8 mg/m^2^ for cycle 1, increasing to 10 mg/m^2^ in subsequent cycles if tolerated. Exploratory studies combining TP300 with other cytotoxics may be appropriate, especially where such combinations have not been feasible with irinotecan due to unacceptable gastrointestinal toxicity.

## Abbreviations

ALT: Alanine amino transferase; AOX1: Aldehyde oxidase 1; AST: Aspartate amino transferase; AUC: The area under the curve; BCEP: Breast cancer resistance protein; CES2: Carboxylesterase 2; C_max_: Maximal plasma concentration; Comet: Cell gel electrophoresis; CTCAE: Common Toxicity Criteria; DLT: Dose-limiting toxicity; EAUC_50_: The AUC associated with 50% of the maximal effect; ECOG: Eastern Cooperative Oncology Group; MTD: Maximum tolerated dose; t_1/2_: Apparent plasma elimination half-life; TM: Tail moment; T_max_: Time of C_max_; Topo-1: Topoisomerase-I; ULN: Upper limit of normal; f_e_: The urinary excretion ratio; PCR: Polymerase chain reaction; SNPs: Single nucleotide polymorphisms.

## Competing interests

DAA, JN, IRJM, DC, JMH, JAH have no competing interests. TS, MA, KJ and MM were employees of the study sponsor. CT and TRJE were both Investigators for the study and members of the project advisory board. CT is also an advisor to some other companies in oncology research.

## Authors’ contributions

CT and TRJE were Investigators and participated in the design of the study, drafting and review of the manuscript. DAA, JN and IRJM were Investigators in the study and drafted the manuscript. DC participated in the study as a Research Nurse, and contributed to review of the manuscript. JMH and JAH conducted and reported the pharmacodynamic assays, and contributed to the writing and review of the manuscript. TS and MA assisted with drafting the manuscript and performed the pharmacokinetic and pharmacogenomic analysis. KJ and MM supported the study and participated in coordination, drafting, review and submission of the manuscript. All authors read and approved the final manuscript.

## Pre-publication history

The pre-publication history for this paper can be accessed here:

http://www.biomedcentral.com/1471-2407/12/536/prepub
